# Retooling Food Service for Early Elementary School Students in Somerville, Massachusetts: The Shape Up Somerville Experience

**Published:** 2009-06-15

**Authors:** Christina D. Economos, Sara C. Folta, Julia Kuder, Valerie Clark, Jeanne Goldberg, Jessica Collins, Mary Jo McLarney, Kozower Claire

**Affiliations:** New Balance Chair in Childhood Nutrition, John Hancock Center for Physical Activity and Nutrition, Friedman School of Nutrition Science and Policy; Tufts University, Boston, Massachusetts; Tufts University, Boston, Massachusetts; Tufts University, Boston, Massachusetts; Tufts University, Boston, Massachusetts; Partners for a Healthier Community, Inc, Springfield, Massachusetts; Somerville Public Schools Food Service Department, Somerville, Massachusetts; Waltham Fields Community Farms, Waltham, Massachusetts

## Abstract

**Background:**

Changes in the school food environment are a logical target to prevent childhood overweight. We describe the food service component of a 2-year research intervention to prevent excess weight gain in children.

**Context:**

The goals of the food service component were to improve the presentation and nutrient quality of school meals and to incorporate more fruits and vegetables into students' diets. The project engaged food service staff, students, parents, teachers, and school leaders to improve school nutrition.

**Methods:**

Modifications addressed needs and barriers identified though dialogue with the food service director, focus groups, key informant interviews, and surveys of school employees, students, and parents and guardians. Attitudes and behavior changes were measured through surveys, direct observation, and sales data.

**Consequences:**

More fruits, vegetables, whole grains, and low-fat dairy products were available during the intervention years; menus and à la carte choices were brought into closer compliance with recommended guidelines for children; attitudes of students, parents and guardians, school faculty, and food service staff improved; and policies related to food service were adopted.

**Interpretation:**

Strategic modification to improve nutrition and increase acceptability of the food served in schools is feasible and sustainable. These results demonstrate that changes to food service can lead to improved nutrient profiles and more favorable attitudes toward food served at school meals. Such changes can help prevent childhood obesity.

## Background

Nearly 31 million children participate in the National School Lunch Program (1) and more than 10 million in the School Breakfast Program (1) every school day, and many students eat most of their daily calories at school (2). School food service departments, which provide a large share of the food that children eat, can play a role in developing children's healthy lifestyles and decreasing the prevalence of overweight and obesity, which has tripled in children aged 6 to 19 years in the last 40 years (3).

School food service departments face many challenges. Financial constraints, inadequate facilities and equipment for food preparation, and a workforce with limited food preparation skills affect the quality, variety, and appeal of food served. Many food service directors rely on commodity foods, which are provided for free by the US Department of Agriculture, to balance tight budgets. Although the nutrition quality of commodity foods offered has improved, school districts often contract with food manufacturers to process the raw commodity foods into ready-to-use convenience products, some of which contribute excess fat, sodium, and sugar. In addition, most food service departments in public school districts are expected to be self-sustaining businesses (4).

Recent interventions demonstrate the feasibility of reducing total fat and saturated fat in school meals and foods available for purchase, increasing the number and variety of fruits and vegetables served, adding more whole grains to school menus, and making sustained changes to à la carte items without long-term adverse effects on sales (5-11). Interventions typically include menu planning, improvements in food purchasing and preparation, promotion of healthier choices, and training and ongoing support for food service staff (10). Although more schools are adopting policies and practices that promote healthy eating in school meal programs, many continue to sell unhealthy foods at fundraisers, school stores, or vending machines or in à la carte lines outside the National School Lunch Program (12).

## Context

To address the growing childhood obesity epidemic, researchers at Tufts University partnered with the city of Somerville, Massachusetts, and Somerville Public Schools to develop and evaluate Shape Up Somerville: Eat Smart, Play Hard (SUS). The goal was to balance early elementary school children's energy intake and expenditure needs by working with key community members to make small changes in the settings children encounter each day. The intervention prevented undesirable weight gain in participating children compared with children from control communities (13). We describe the development, implementation, and evaluation of the school food service component, a major component of SUS.

Somerville, a diverse, urban city north of Boston, has a median household income of $46,315; 13% of families with children under age 18 live below the poverty line (14). Close to 50% of students belong to racial/ethnic minority groups, and nearly 50% speak English as a second language (15). More than 60% are eligible for free or reduced-price lunches (15). Breakfast is free for all, regardless of income. The Somerville Food Service Department provides approximately 1,800 breakfasts, 3,600 lunches, and 650 after-school snacks daily. The food service system is centralized, and individual schools have limited food preparation facilities.

All elementary school children (kindergarten through eighth grade) were exposed to the food service intervention, but individual-level evaluation included only those in grades 1-3 and their parents or guardians who consented to participate in SUS. The intervention was planned during the 2002-2003 school year and executed in 2003-2004. It was expanded and further monitored for sustainability in 2004-2005.

## Methods

So that we could understand the target community's perspective and establish common goals to facilitate the design and implementation of the intervention, we communicated with the food service director and held focus groups and interviews with school employees, students, and parents and guardians. During the summer of 2003, focus groups of 6 to 8 people each provided information about dietary behaviors, feelings about school food, and feedback on potential initiatives. A total of 13 focus groups were held, 3 with food service staff, 5 with students, and 5 with parents. Participants included 40 first- through third-grade students, 39 parents, and 24 food service staff. Sessions with children and parents were held in English, Portuguese, or Spanish. Nineteen key informant interviews with school representatives and community members provided feedback on community priorities and potential nutrition initiatives. Investigators combined data from these 2 sources with information obtained from the food service director to design and implement the food service intervention.

Three main components made up the school food service intervention: school meal changes, professional development and capacity building, and communication strategies (Figure).

**Figure.**
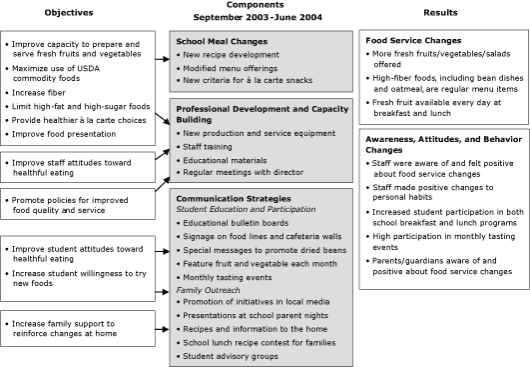
School food service intervention framework — Somerville, Massachusetts.

### School meal changes

Primary objectives of school meal changes were to improve the presentation and nutrient quality of school meals and to introduce and incorporate fresh fruits and vegetables into students' diets. Parents who participated in focus groups expressed an interest in healthier school meals, specifically increasing fresh produce, decreasing processed foods, and decreasing high-sugar cereals and snacks. An audit of menus and recipes identified opportunities for healthier options. Research staff engaged students and food service employees to generate ideas, develop recipes, sample items, and provide feedback. Food service cooks adapted approved recipes for large-scale production.

Menu adjustments included increasing the quantity and variety of produce at breakfast and lunch and ensuring that at least some breakfast cereals had at least 1 g fiber and less than 10 g sugar per serving. The daily mix of cold cereal choices at breakfast included at least 2 high-fiber cereals Monday through Thursday and exclusively high-fiber cereals on Fridays. At the time of the study, fewer cereals, especially cereals marketed to children, contained fiber or were made with whole grains. Although a few low-sugar cereals containing fiber were available in the district before the intervention, cafeteria employees did not offer them at breakfast because they felt the healthier cereals were unpopular with students. Study staff and the food service manager agreed to begin offering only healthy cereals on Fridays to minimize any negative financial effects because breakfast participation was lowest on Friday. Each meal included fresh fruit. Side salads were offered once per week, and entrée salads were offered 3 times per week. Choices of new à la carte items met the criteria from the Berkeley, California, school district (4); these choices were adjusted to meet Massachusetts Action for Healthy Kids guidelines when they became available (16). Ice cream was sold only on Fridays.

The district hired school breakfast coordinators at 3 elementary schools to supervise and model healthy eating for children participating in the School Breakfast Program. A local vendor donated fruits and vegetables that were featured in school meals and at tasting events.

### Professional development and capacity building

Focus groups identified inadequate or outdated equipment as a barrier to preparing and serving healthy foods. Therefore, the study purchased equipment to improve food preparation, service, and presentation. Items that were purchased included essential tools such as sheet pans, knives, cutting boards, measuring utensils, thermometers, food processors, and electric peelers. A commercial convection oven for the main kitchen, display coolers, and large fruit bowls to improve appearance of the serving line were also purchased. The total cost of the equipment was $34,351, or $2.38 per child in kindergarten through eighth grade.

Food service staff were trained throughout the year. Outside professionals led workshops on various skills, with an emphasis on knife skills and fresh vegetable preparation. SUS staff led nutrition workshops that focused on practical information to increase knowledge about nutrition and physical activity, promote healthy diets, and encourage positive role modeling for students. Monthly small-group classes with kitchen leaders from each school included education about why new foods were being introduced and demonstrations of preparation techniques. SUS staff and the food service director met weekly to assess the intervention and address problems.

### Communication strategies

SUS staff collaborated with principals, teachers, and local media outlets to communicate with students and to encourage them to try different foods. In addition to monthly tasting events at which students were offered samples of the fruit or vegetable of the month, these items were served weekly throughout the month in school meals. The food service department developed new recipes using these foods for the meal programs. Posters and table tents with nutrition information, motivational messages, and fun facts about healthful foods were displayed in the cafeteria. Principals and teachers helped promote the program through school announcements and related SUS classroom lessons. Several schools played a series of prerecorded messages promoting new vegetarian bean dishes on the school announcement system (17). During the year, children tasted 11 different fruits and vegetables.

Information obtained in focus groups indicated a need for communication through multiple channels to encourage family participation. Notices about upcoming school food events were sent home with students. The food service department mailed 9 newsletters, with new recipes and nutrition information, to participants' homes. The local newspaper printed and cable television channels aired school menus. SUS personnel and the food service director attended parent-teacher conference nights to raise awareness of and answer questions about food service changes. Families were invited to enter healthy lunch ideas into a schoolwide recipe contest.

### Evaluation

We assessed the effect of school meal changes by documenting initiatives, direct observation, surveys, tracking meal participation, and assessing costs and revenue. SUS interviewers administered surveys to assess changes in student attitudes about school meals before and after the first intervention year (13). We analyzed within-group changes by using paired *t* tests and between-group differences by using independent-samples *t* tests; all analyses were conducted with SPSS version 14 (SPSS, Inc, Chicago, Illinois). Production records were used to track changes in children's choices of items offered, and sales records were used to assess changes in à la carte purchases.

In September 2003, before the intervention began, food service employees completed a 75-question survey to gauge their overall impression of school food service and the proposed changes to it and to assess their vegetable preparation skills and knowledge. In June 2004, a 49-item survey asked their opinions of changes and initiatives throughout the year and the effect of the changes on meal quality, student satisfaction, food service workload, and personal habits. Both surveys were formatted with multiple choice, yes/no, and Likert-type scale items. Investigators surveyed food service employees of schools that received new equipment to evaluate its effect on food preparation, serving time, and presentation. Midway through the school year (January 2004) kitchen leaders and school principals completed an 8-question survey to assess their perceptions of monthly tasting events and to identify problems.

Tasting events were evaluated in all elementary schools by students, school personnel, and parents. Students voted on whether they liked the items served and would choose them if offered as part of school lunch. At the final tasting (June 2004), they received a list of all foods tasted throughout the year and were asked to circle their 3 favorites. Investigators mailed a survey, translated into Haitian Creole, Portuguese, and Spanish, to parents and guardians to assess their awareness of school food service events and changes. Reminder postcards were sent 1 week later. Nonrespondents were mailed a second copy 2 weeks later.

## Consequences

### School meal changes

Participation in school breakfast and lunch increased 3% during the 2003-2004 school year. During that year, fresh produce expenditures totaled $117,000, an increase of $27,000 from the previous year. Fresh produce donations, valued at $35,000, helped to offset the increased cost. Nonproduce food costs were similar to previous years. Students' actual consumption of produce offered is the purview of another manuscript. During the second year (2004-2005), when no donations were provided, school food service spent approximately $143,000 on fresh produce to meet increased student demand. Improved control of food costs, increased revenue from increased meal participation, and decreased waste due to more accurate forecasting of student meal choice provided the funds to continue to offer fresh produce after the donation period ended.

Menu changes during the first intervention year were carefully documented (Table). When only high-fiber, low-sugar cereals were offered on Fridays, the percentage of children who chose cold cereal (28%) was lower than on days when 2 healthy cereals were offered along with the standard mix of high-sugar, low-fiber cereals (41%). Side salads were served 1 day per week compared with less than once per week in the preintervention year. Availability of fresh fruits at breakfast and lunch increased from twice per week to 5 times per week during the intervention year. Oatmeal was served an average of 2.7 times per month and more frequently in winter, compared with not at all before the intervention. Vegetarian bean entrées were served 1-2 times per month. Children chose a vegetarian bean entrée over the standard competing entrée (hot dog) 24% of the time (17). Total sales, including à la carte snacks, milk, paid student meals, and teacher purchases decreased when the new mix was introduced (September to December 2003) but returned to preintervention levels in February 2004 and were sustained throughout the remainder of the school year.

### Professional development and capacity building

A priority of this study was to identify intervention components that could become institutionalized through policies. Food service policy changes included serving daily at least 2 low-sugar, high-fiber cereals at breakfast and fresh fruit at breakfast and lunch. The school system adopted the Massachusetts Action for Healthy Kids guidelines for à la carte items (16). Ongoing health education for food service personnel must be delivered annually.

Other systemwide policies also had a direct effect on school food service. The district wellness policy encourages schools to allow students more time for lunch and to provide recess before lunch for all students through eighth grade. The policy also encourages schools to hold fundraisers that do not involve food or to choose foods sold from a recommended list. Food fundraising items sold during school hours are offered only after the school lunch is over.

The September 2003 preimplementation food service survey (N = 15, 83% response rate) showed that 5 personnel believed that the children liked vegetables and 10 believed they liked fruits. Three thought the children were open to trying new foods, 11 believed they could influence children to choose more fruits and vegetables at lunch, and 12 believed they could influence them to eat them. Most personnel rated proposed promotional activities highly.

All food service workers who completed the year-end survey in June 2004 (N = 20, 71% response rate) agreed that school lunch had improved since September 2003. Most thought that students liked the fruits served at lunch, but only approximately one-third thought that they liked the vegetables. Food service staff thought the changes required more effort on their part but were optimistic about being involved in the efforts.

All food service staff (N = 13) from schools that received new equipment reported in the year-end survey that the equipment enabled them to better prepare and serve meals. Specifically, 8 reported that it speeded preparation time, 7 said that it speeded serving time, 9 felt that it improved food presentation, and 6 said that it improved food quality. Most of those who did not respond positively said they were unsure whether the new equipment helped.

On the year-end survey, food service workers reported changes in their personal eating habits: 15 were trying to eat more vegetables, 13 were trying to eat fewer high-fat foods, 10 were trying to eat fewer snacks, 9 were trying to eat more whole grains, and 8 were trying to drink fewer artificially sweetened beverages. As reasons for making these changes, 10 cited wanting to lose weight, 13 to improve their health, and 9 to reduce their chance of illness.

### Communication strategies

More than 80% of students participated in the tasting events. At the final taste test, children in grades 1 through 3 (N = 869) voted on their top 3 favorites. Although the 3 most popular items were fruits, 17%, 16%, and 15% cast 1 of their 3 votes for broccoli, spinach, and cherry tomatoes, respectively.

The midyear food service survey found that 90% of kitchen leaders (N = 10) and all school principals (N = 8) believed that students enjoyed the tastings; all kitchen supervisors and 7 principals thought that the events encouraged students to try new foods. Nine leaders and 7 principals thought that the events encouraged students to eat more produce.

Data from after the first year of the intervention revealed a significantly reduced preference for school lunch in both the intervention and combined control communities (*P* = .001 and .005, respectively). The difference between the groups was not significant (*P* = .19), even though control communities received no food service intervention. The observation that preference for school lunch did not decline more in intervention than in control schools is encouraging, since Somerville school lunches became more healthful after the intervention.

In the 2004 end-of-year surveys sent to parents and guardians (N = 216, 44% response rate), 54% said they were aware that more fresh produce was offered as side dishes, and 52% were aware that cafeterias were selling healthful snacks. Sixty-six percent said they were aware of fruit and vegetable taste tests.

## Interpretation

Our study adds to the evidence that schools can make positive menu changes that meet federal standards, even in the face of many constraints (10,11,18). Because a large percentage of students receive free or reduced-price school meals, Somerville was an ideal city to initiate an intervention to improve food service in the context of serious cost limitations. In Somerville, average food costs were $0.85 per child per meal from 2002 through 2004. Future analyses and studies will need to examine whether menu changes in school food service affect students' diets.

Several factors were critical to success. Commitment of the food service director and managers was essential. Visible, comprehensive outreach to school and community members about program changes increased cooperation.

The food service staff training and education program focused on skill building, use of new equipment, and personal health. That focus led to buy-in from the group that was most critical to success but somewhat resistant to change at the outset. Negotiations with the union representing the food service employees allowed for production changes in favor of increased scratch cooking and use of fresh fruits and vegetables to be institutionalized.

The lack of cash registers at several schools made accurate tracking of the financial effect of the intervention a challenge. A computerized cash register system has now been installed at all schools and improves the ability to analyze sales data.

We continued to address barriers encountered after 1 intervention year. To further reduce staff resistance to changes, we offered additional wellness opportunities such as motivational workshops, yoga, and strength-training programs. In retrospect, more personal wellness opportunities for food service staff at the beginning of the intervention might have helped motivate them to improve attitudes and enthusiasm about the changes due to the intervention. To build on improvements in meal quality and staff capacity, we obtained grant funding to hire a consultant chef to provide consistent on-site training, oversight of meal preparation, and reinforcement. Including a chef in the intervention plan should be considered.

This project overcame barriers common to school food service systems and succeeded in implementing changes. Unique intervention features came directly from needs identified through extensive formative research. Student participation increased, nutrient profiles of à la carte items improved, and the amount of fruits, vegetables, whole grains, and low-fat menu items increased. As reported in other studies, food service training was critical to success.

## Figures and Tables

**Table.** Menu Changes in Somerville Public Schools Resulting From the Shape Up Somerville Food Service Intervention, 2003-2004 School Year

Menu Item	Frequency per Week		Goal

	Preintervention	Intervention	
**Breakfast**			
Fresh fruits	2	5	Increase produce intake
Low-sugar, high-fiber cereals	0^a^	Monday-Thursday, 2 options daily; sole option on Friday	Decrease sugar intake, increase fiber intake
Instant oatmeal (November-March)	0	2	Increase fiber intake, increase whole grain intake
**Lunch**			
Fresh fruits	2	5	Increase produce intake
Entrée salads (grades 4-8)	2	3	Increase produce intake, decrease fat intake
Side salads (grades 1-3)	<1	1	Increase produce intake, decrease fat intake
Vegetarian bean entrées	0	0.25-0.50	Increase fiber intake, decrease fat intake
**À la carte**			
Ice cream	5	1	Decrease fat intake
Snacks	5 (regular chips, large cookies, and ice cream)	5 (baked chips, hot pretzels, popcorn, yogurt, granola bars)	Decrease fat intake, decrease sugar intake

a These cereals were available for schools to purchase but not routinely offered to students because of perceived student preference for high-sugar cereals.
